# F-type complete mitochondrial genomes of two *Hyriposis* species, *H. schlegelii* and *H. cumingii* (Bivalvia; Unionoida; Unionidae) from Lake Kasumigaura, Japan, and its phylogenetic analysis

**DOI:** 10.1080/23802359.2020.1730727

**Published:** 2020-02-27

**Authors:** Yohei Fukata, Masayuki Iigo

**Affiliations:** a Department of Applied Biological Chemistry, School of Agriculture, Utsunomiya University, Tochigi, Japan;; b Department of Applied Life Science, United Graduate School of Agricultural Science, Tokyo University of Agriculture and Technology, Tokyo, Japan;; c Center for Bioscience Research and Education, Utsunomiya University, Tochigi, Japan;; d Center for Optical Research and Education, Utsunomiya University, Tochigi, Japan;; e Center for Weed and Wildlife Management, Utsunomiya University, Tochigi, Japan

**Keywords:** *Hyriposis schlegelii*, *H. cumingii*, Unioninae, Illumina sequence, Molecular phylogenetic analysis

## Abstract

We have sequenced the female type (F-type) complete mitochondrial genomes of two *Hyriposis* species, *H. schlegelii* and *H. cumingii* (Gonideinae, Unionidae, Unionida, Bivalvia) from Lake Kasumigaura, Japan, and inferred the Unioninae phylogeny. Complete mitochondrial genomes (*H. schlegelii*, 15,954 bp, LC498622; *H. cumingii*, 15,961 bp, LC498621) contain 13 protein-coding genes (PCGs), 2 rRNA genes, and 22 tRNA genes. Molecular phylogenetic analyses using the 13 PCGs including the two species were performed. This study should be basic data to investigate the evolution of Gonideinae and genetic diversity of *Hyriposis* species in local populations.

*Hyriopsis schlegelii* is an endangered species, endemic to Lake Biwa, Japan. Since the 1930s, it was transplanted to Lake Kasumigaura, Japan. *Hyriopsis cumingii* inhabits in China, and produces freshwater pearls (Wei et al. 2016). This species was brought to Lake Kasumigaura from the 1970s to 1980s. Since the two species inhabited sympatrically, hybridization between the two has been suspected (Shirai et al. 2010).

To obtain molecular basis for the conservation of the two species in Lake Kasumigaura, we have sequenced complete mitochondrial genomes of *H. schlegelii* and *H. cumingii* collected at Lake Kasumigaura and molecular phylogenetic analyses of Gonideinae were performed.

*Hyriopsis schlegelii* and *H. cumingii* (#UU-SBD-Unio-10 and 11, respectively) were collected at Lake Kasumigaura, Japan (N35.992, E140.349). DNA extraction and sequence analyses were performed as described by Fukata and Iigo (2019). Local BLAST search using the mitochondrial genome of *H. schlegelii* (AP018551) and *H. cumingii* (HM347668) identified the F-type complete mitochondrial genomes of the two species (circular; *H. schlegelii*: 15,954 bp: *H. cumingii*, 15,961 bp). The complete mitogenomes contain 13 protein-coding genes (13PCGs), 2 rRNA genes, and 22 tRNAs. The sequences have been submitted to DDBJ/EMBL/Genbank with accession numbers LC498622 (*H. schlegelii*) and LC498621 (*H. cumingii*).

Male and female mitogenomes of Gonideinae are known to have different gene orders (Breton et al. 2009). The addition of M-type sequences to the traditional F-type mitogenomes could often result in more robust evolutionary relationships than those provided by the F-type only (Walker et al. 2006). As expected, molecular phylogenetic tree constructed by MEGA X (Kumar et al. 2018) using the 13 PCGs of Gonideinae revealed two main clades (F-type and M-type; Figure 1). Based on the tree, the three mitogenomes (NC_015110, HQ641407 and NC_011763) seem to have been misidentified: NC_011763 (*H*. *cumingii*) might be *H. schlegelii*; NC_015110 and HQ641407 (both registered as *H. schlegelii*) might be the F-type mitogenomes of a new *Hyriposis* species (identity of nucleotides and deduced amino acid sequences to the F-type *H*. *cumingii* and *H. schlegelii* range from 94.0 to 94.8 and 89.3 to 90.6%, respectively).

**Figure 1.**
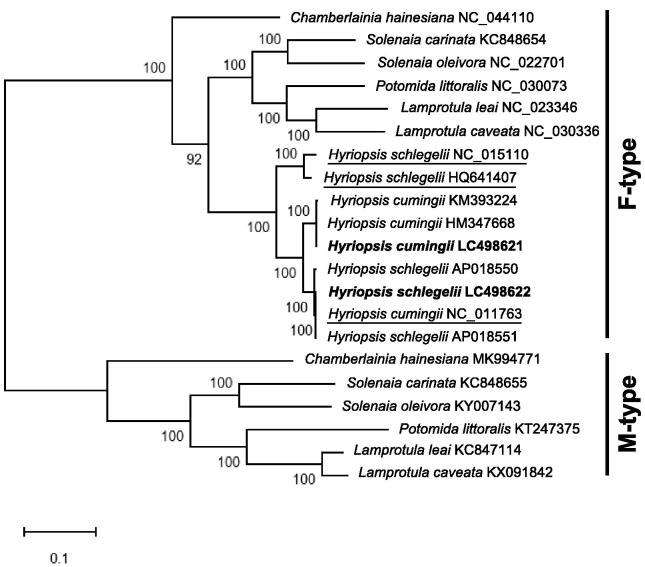
Molecular phylogenetic tree (Maximum likelihood method) using 13 protein-coding genes of Gonideinae including *H. schlegelii* and *H. cumingii* from Lake Kasumigaura, Japan. The numbers above the branch meant bootstrap value (1000 replicates). Leaf names were presented as species names and accession number. Sequences determined in this study (LC498621 and LC498622) are in bold. Sequences thought to be misidentified are underlined.

This research would provide valuable information for investigating the evolution of Gonideinae and the genetic diversity of local populations of *Hyriposis* species.
